# Macrophage TGF-**β** signaling is critical for wound healing with heterotopic ossification after trauma

**DOI:** 10.1172/jci.insight.144925

**Published:** 2022-10-24

**Authors:** Nicole K. Patel, Johanna H. Nunez, Michael Sorkin, Simone Marini, Chase A. Pagani, Amy L. Strong, Charles D. Hwang, Shuli Li, Karthik R. Padmanabhan, Ravi Kumar, Alec C. Bancroft, Joey A. Greenstein, Reagan Nelson, Husain A. Rasheed, Nicholas Livingston, Kaetlin Vasquez, Amanda K. Huber, Benjamin Levi

**Affiliations:** 1 Section of Plastic Surgery, Department of Surgery, University of Michigan Medical School, Ann Arbor, Michigan, USA.; 2 Department of Surgery, UT Southwestern Medical Center, Dallas, Texas, USA.; 3 Department of Epidemiology and Emerging Pathogens Institute, University of Florida, Gainesville, Florida, USA.; 4 Epigenomics Core, University of Michigan Medical School, Ann Arbor, Michigan, USA.; 5 Acceleron Pharma, Inc., Cambridge, Massachusetts, USA.

**Keywords:** Bone Biology, Immunology, Growth factors, Macrophages

## Abstract

Transforming growth factor–β1 (TGF-β1) plays a central role in normal and aberrant wound healing, but the precise mechanism in the local environment remains elusive. Here, using a mouse model of aberrant wound healing resulting in heterotopic ossification (HO) after traumatic injury, we find autocrine TGF-β1 signaling in macrophages, and not mesenchymal stem/progenitor cells, is critical in HO formation. In-depth single-cell transcriptomic and epigenomic analyses in combination with immunostaining of cells from the injury site demonstrated increased TGF-β1 signaling in early infiltrating macrophages, with open chromatin regions in TGF-β1–stimulated genes at binding sites specific for transcription factors of activated TGF-β1 (SMAD2/3). Genetic deletion of TGF-β1 receptor type 1 (*Tgfbr1*; *Alk5*), in macrophages, resulted in increased HO, with a trend toward decreased tendinous HO. To bypass the effect seen by altering the receptor, we administered a systemic treatment with TGF-β1/3 ligand trap TGF-βRII-Fc, which resulted in decreased HO formation and a delay in macrophage infiltration to the injury site. Overall, our data support the role of the TGF-β1/ALK5 signaling pathway in HO.

## Introduction

Transforming growth factor–beta (TGF-β) signaling is essential for normal tissue-specific regeneration and aberrant wound healing. The response to injury following a traumatic event can be divided into hemostasis, inflammation, proliferation, maturation, and remodeling (1). In each stage of healing, TGF-β plays a number of critical roles that vary in context and in a cell type–dependent manner, including regulation of cell proliferation, differentiation, migration, invasion, and chemotaxis of fibrotic and immune cells (2, 3). Specifically, in normal fracture healing, TGF-β plays a pivotal role by enhancing the proliferation and differentiation of mesenchymal stem/progenitor cells (MPCs), increasing the production of extracellular matrix, and acting as a chemoattractant to osteoblasts (4). TGF-β has also been shown to play a key role in cartilage formation and increases the formation of callus and bone strength (5). In vivo experiments have demonstrated accelerated fracture healing and enhanced bone remodeling with TGF-β (6, 7). Similarly, aberrant ectopic bone formation or heterotopic ossification (HO) following trauma injury or hip arthroplasty has been shown to have increased TGF-β expression near the injury or surgical site (8–10). Overexpression of TGF-β in tendon has been shown to induce spontaneous HO, whereas TGF-β neutralizing antibody attenuates ectopic bone formation in traumatic mouse models (9). These findings support the critical role of TGF-β in both normal and abnormal wound healing in the bone, but the precise mechanism by which TGF-β acts on the surrounding local environment and myeloid cells remains to be fully elucidated.

Both infiltrating immune cells, specifically monocytes and macrophages, and MPCs participate in the process of both normal and aberrant bone formation after injury or trauma (10, 11). Specifically, TGF-β1 produced by macrophages has been shown to stimulate chondrogenesis in MPCs (10, 12), which is a fundamental process for endochondral ossification. In addition to chondrogenesis, TGF-β1 signaling in macrophages has been shown to modify immunogenicity through altering cell polarization and migration, which has been shown to further promote bone formation (13–15). Activation by TGF-β1 results in heterodimerization of TGF-βRII with TGF-βRI, also known as activin receptor-like kinase 5 (ALK5), and allows for downstream canonical TGF-β signaling, which involves phosphorylation of SMAD2/3 and translocation of phosphorylated (p-) SMAD2/3 to the nucleus to activate gene transcription (16). *Alk5* deletion in monocytes has been shown to inhibit proinflammatory and promote antiinflammatory macrophage markers of expression (17). Gong et al. demonstrated that while knocking out TGF-βRII in hematopoietic cells did not affect the efferocytotic ability of macrophages, it resulted in the inability of macrophages to upregulate M2-polarized genes (18). Our group and others have shown that TGF-β1 expression, specifically by myeloid cells, is critical to HO formation after traumatic injury (9, 10). In the mouse model for traumatic HO, deletion of macrophage *Tgfb1* resulted in decreased HO formation. Furthermore, treatment with a CD47 activating peptide decreased macrophage TGF-β1 expression, skewed macrophage polarization away from an M2 phenotype toward a more resident macrophage phenotype, and resulted in decreased HO (10). However, it is unknown whether the TGF-β1 produced by macrophages further alters the macrophage phenotype and function or affects the local wound environment to alter matrix production or differentiation of MPCs (19, 20).

In the current study, we investigated the impact of TGF-β signaling in macrophages and the MPCs in the local wound environment. Utilizing a mouse model of HO and single-cell transcriptomic and epigenomic analyses, we found specific increases in TGF-β–stimulated gene expression as well as open chromatin regions at p-SMAD2/3 binding sites in TGF-β1–stimulated genes in macrophages and MPCs. Overall, the findings are suggestive of an autocrine effect of TGF-β1 in macrophages. Targeted deletion of *Alk5* in macrophages (*LysMCre*) further corroborated the autocrine effect of TGF-β receptor signaling on macrophages, which was not observed with deletion of *Alk5* in MPCs (*Hoxa11CreER^T2^*). Due to some additional effects seen in macrophage receptor deletion, we opted to treat upstream of the receptor by targeting the TGF-β ligands. Treating injured mice systemically with a ligand trap, TGF-βRII-Fc, which blocks both TGF-β1 and TGF-β3 ligand, resulted in attenuated HO formation and in delayed macrophage infiltration. Taken together, these findings suggest that macrophage ALK5 signaling potentiates aberrant bone formation and that pharmacological inhibition of TGF-β1 with the ligand trap TGF-βRII-Fc is a potential therapeutic agent to prevent HO.

## Results

### Increased canonical TGF-β signaling at the HO site after burn and tenotomy.

**Figure 1:**
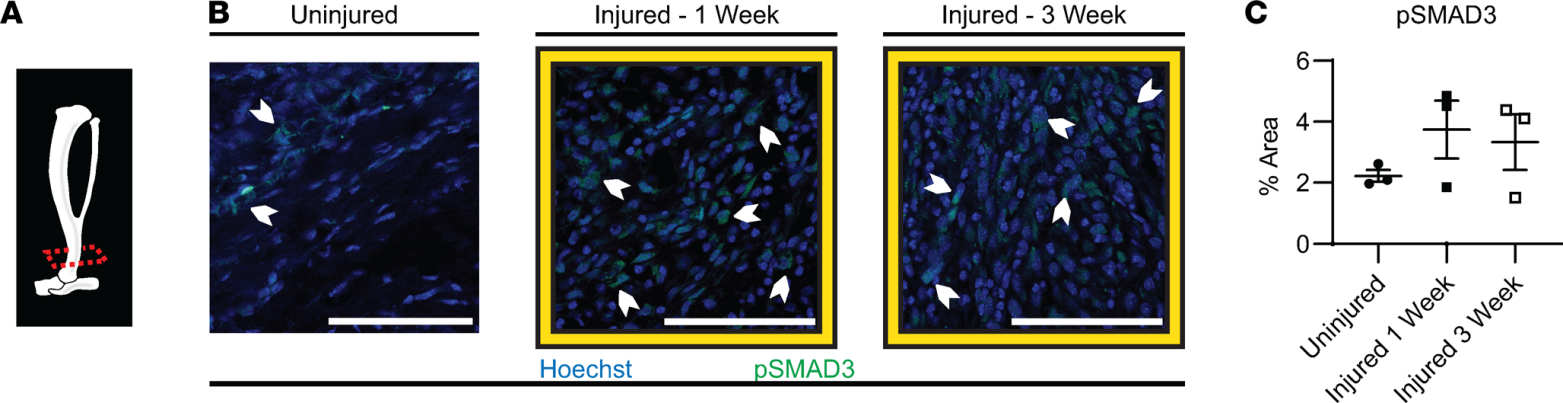
Canonical TGF-β signaling in the mouse distal hind limb where HO forms. Immunofluorescence (IF) images, with (**A**) micro computed tomography (MicroCT) graphic showing the histology section level used at time points indicated. (**B**) Effects of TGF-β signaling by proxy of p-SMAD3 (green) and nuclear Hoechst (blue) in uninjured, 1 week after injury, and 3 weeks after injury. White chevrons point out cells as examples of positive p-SMAD3 staining. Scale bars represent 100 μm. (**C**) Quantification for percentage area of p-SMAD3 at uninjured (*n* = 3/group, 2–3 images/*n* mice) 1 week postinjury (*n* = 3/group, 3 images/*n* mice), 3 weeks postinjury (*n* = 3/group, 3 images/*n* mice). Error bars represent mean ± SEM.

**Figure 2:**
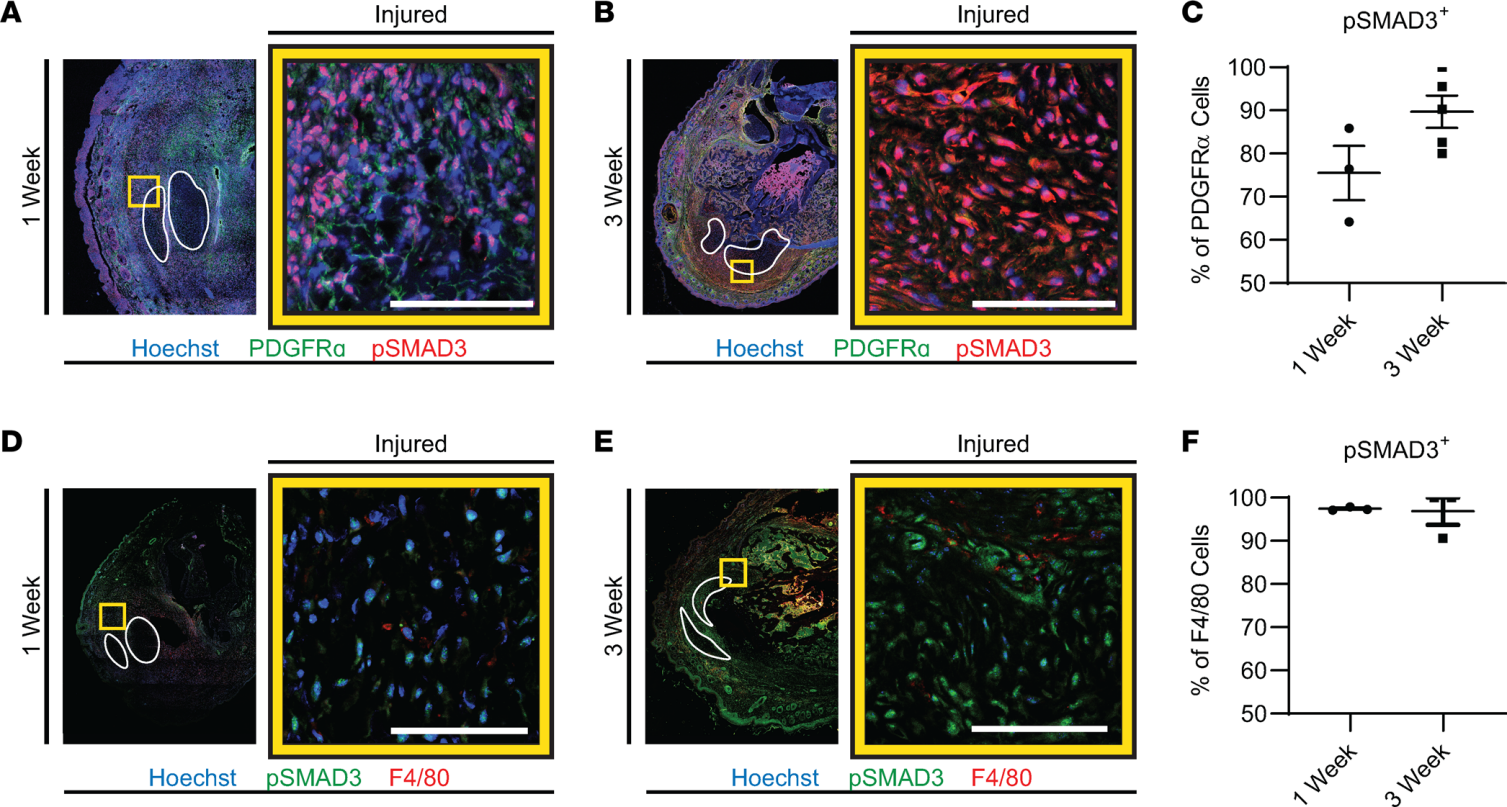
Canonical TGF-β signaling in MPCs and macrophages. IF images, merged tile scan with the tendons outlined in white and the yellow box showing the 63× zoomed-in image to the right. Scale bars represent 100 μm and for quantifications, error bars represent mean ± SEM or median ± interquartile range. (**A**) IF images for p-SMAD3 (red) in PDGFRα^+^ cells (green) and nuclear Hoechst (blue) for 1 week and (**B**) 3 weeks (*n* = 3 and 5/group), with (**C**) quantifications. (**D**) IF images are of p-SMAD3 (green) in F4/80^+^ cells (red) and nuclear Hoechst (blue) with quantifications for 1 week and (**E**) 3 weeks (*n* = 3/group), with (**F**) quantification.

Increased TGF-β activity has been shown to be present in human HO tissue (9). When TGF-β1 binds to its receptor, SMAD2/3 is phosphorylated and translocates to the nucleus, which then activates target genes responsible for a variety of cell-specific functions (21–39). We therefore examined the canonical signaling pathway in mice by assessing percentage area of p-SMAD3 in HO anlagen at 1 week and 3 weeks after injury. Similar to what has been previously described, we found a trend toward greater p-SMAD3 staining from the HO injury site after 1 week, 3.7% ± 0.9%, and at 3 weeks, 3.3%± 0.9%, compared with uninjured hind limb, 2.3% ± 0.2% (Figure 1 and Supplemental Figure 1; supplemental material available online with this article; https://doi.org/10.1172/jci.insight.144925DS1). Both MPCs and macrophages express the receptor for and can respond to TGF-β1; therefore, we analyzed TGF-β1 signaling by p-SMAD3 specifically in these populations. MPCs, marked by PDGFRα, had an increased percentage of cells that were p-SMAD3 positive at 3 weeks (89.7% ± 3.8%) compared with 1 week (75.5% ± 6.3%, *P* = 0.0819) (Figure 2, A–C, and Supplemental Figure 2). Alternatively, percentages of the infiltrating tissue macrophages that were p-SMAD3 positive at 1 week and 3 weeks after injury were similar and very high at 97.4% ± 0.2% and 96.9% ± 3.1%, respectively (*P* = 0.8657) (Figure 2, D–F, and Supplemental Figure 3, A–C). Increased p-SMAD3 in MPCs and the near-ubiquitous signaling in macrophages following injury suggest TGF-β signaling is present or upregulated in these cell populations in our trauma-induced HO model early on, before the process of cartilage formation has started.

### Changes in TGF-β–stimulated genes in trauma-induced HO.

**Figure 3:**
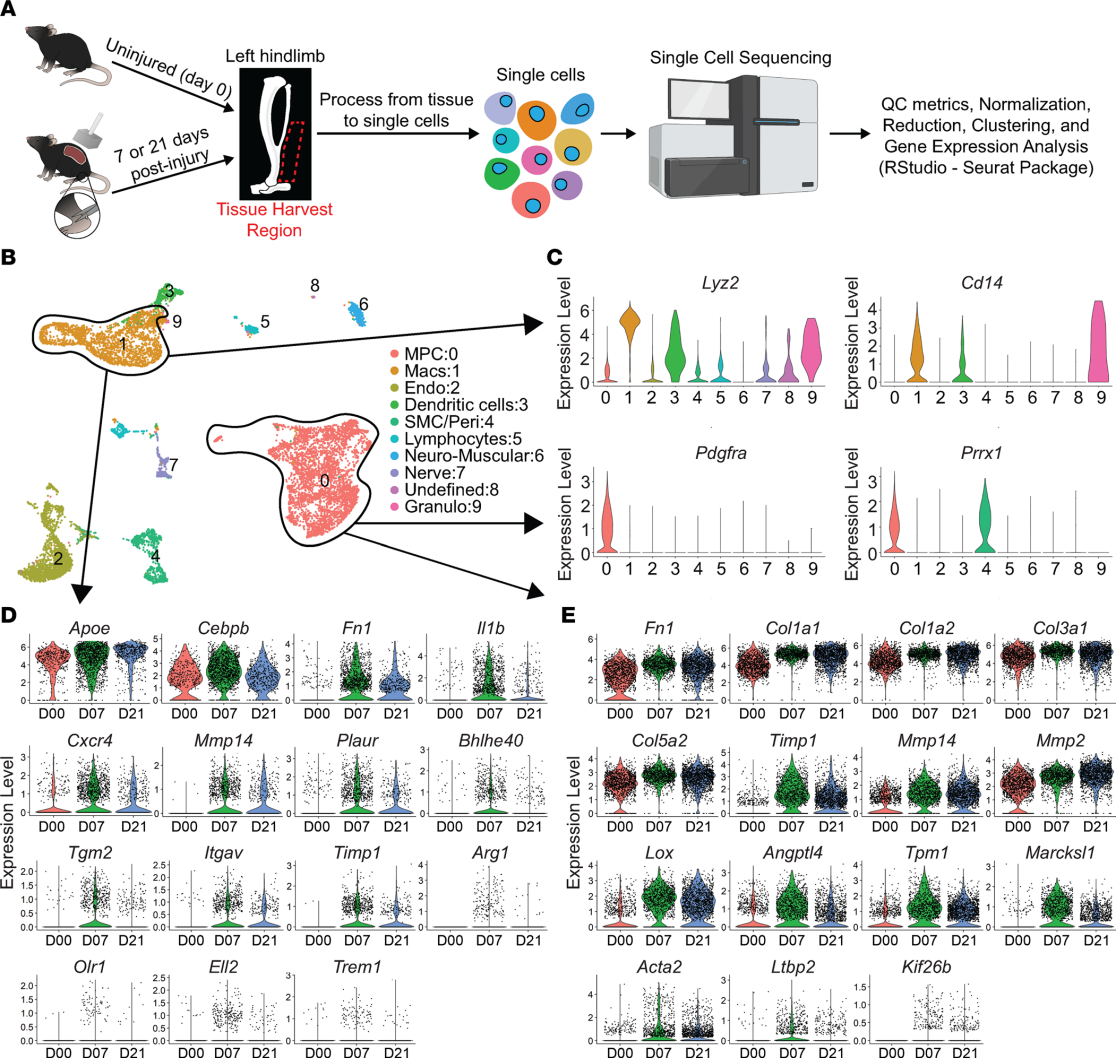
Single-cell sequencing showing change in genes regulated by TGF-β signaling. (**A**) Overview of tissue to obtain results from scRNA-Seq. (**B**) Uniform manifold approximation and projection (UMAP) plot with all time points clustered and legend to the right of the plot. The MPC and macrophage (Mac) clusters are circled. Endo, endothelial; SMC/peri, smooth muscle cell/pericyte; Granulo, granulocyte. (**C**) Violin plots of genes marking MPCs and Macs. (**D**) Genes regulated by TGF-β in Macs from day 0 (uninjured), day 7, and day 21. (**E**) Genes regulated by TGF-β in MPCs from day 0 (uninjured), day 7, and day 21.

**Figure 4:**
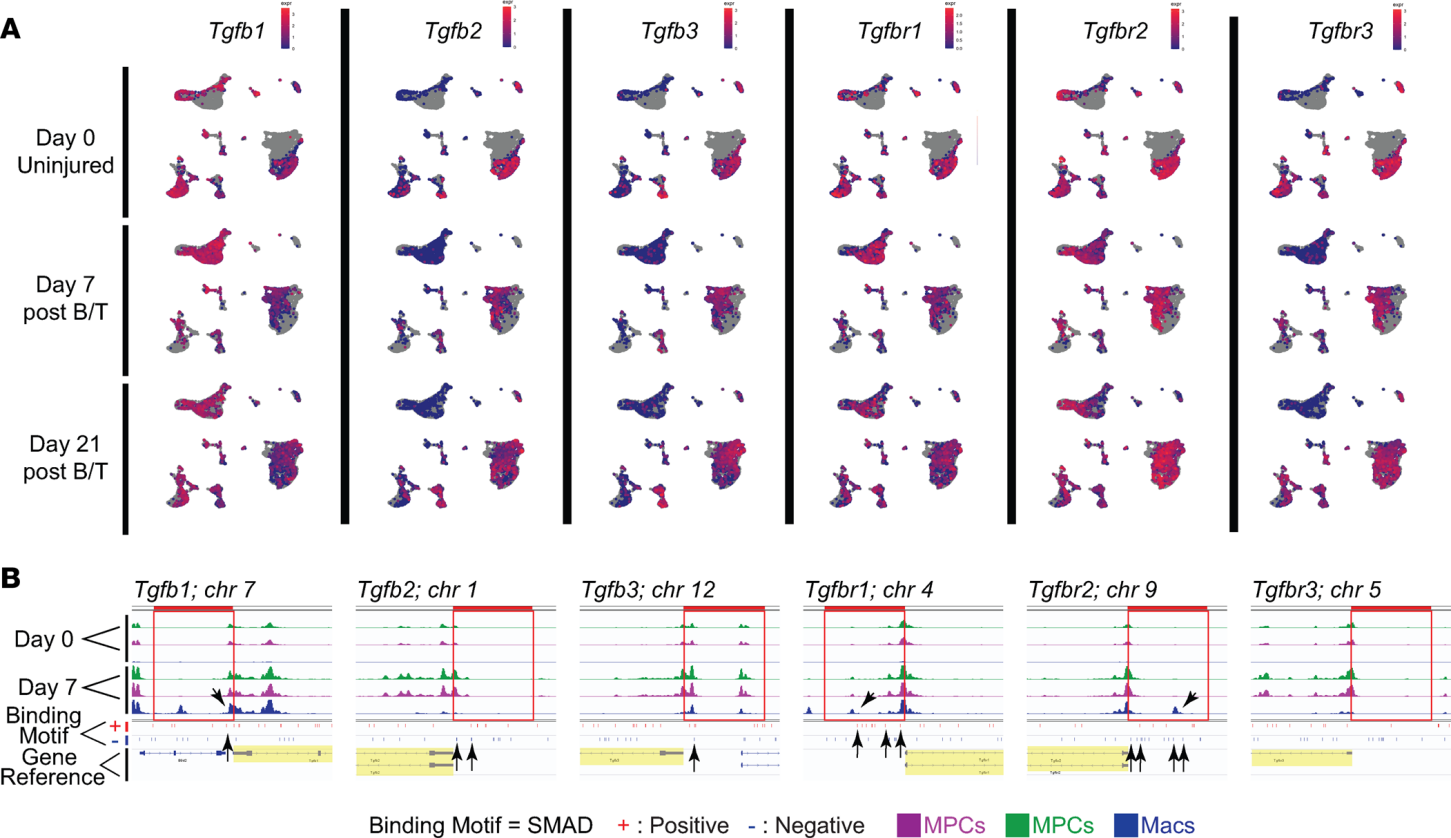
TGF-β downstream signaling in MPCs and Macs with change in open chromatin for TGF-β ligands and receptors. (**A**) UMAP plots of TGF-β ligand and receptor genes for days 0, 7, and 21. (**B**) Images of open chromatin in promoter region by snATAC-Seq associated with SMAD binding regions for TGF-β ligand and receptor genes stimulated by TGF-β. The tracks shown are color-coded by their cluster identity such that green and magenta are MPCs and blue are Macs. All track data are presented in the range of 0 to 800. For each reference gene, the 5 kb upstream promoter region is indicated by a red box overlying the tracks. The reference gene is highlighted yellow. Black arrows are used to assist in indicating the regions of open chromatin at or near SMAD binding sites.

Our group and others have shown TGF-β1, specifically expressed by myeloid cells, is critical to HO formation after traumatic injury (9, 10). While we see increased TGF-β signaling at the HO anlagen, it is unknown whether TGF-β1 expressed by macrophages during a traumatic injury acts in an autocrine or paracrine fashion and in which cell type this signaling is necessary for the formation of HO. To begin to assess this, we used single-cell RNA sequencing (scRNA-Seq) performed on cells harvested from the hind limbs at days 0 (uninjured), 7, and 21 after burn/tenotomy (NCBI Gene Expression Omnibus [GEO] GSE126060). Workflow for scRNA-Seq is shown in Figure 3A. Clustering was done as previously characterized. MPC clusters were identified based on their expression of *Pdgfrα* and *Prrx1* (Figure 3, B and C) (40). Mac clusters were identified based on high expression of known markers *Lyz2* and *Cd14* (Figure 3, B and C). The MPC and Mac clusters were assessed for genes known to be transcribed upon TGF-β stimulation. When we looked at these TGF-β–stimulated genes in the Macs, we found 15 genes known to be controlled through TGF-β signaling, many important in the immune function of Macs, increased at day 7 compared with uninjured, including *Apoe*, *Cebpb*, *Fn1*, *Il1b*, *Cxcr4*, *Mmp14*, *Plaur*, *Bhlhe40*, *Tgm2*, *Itgav*, *Timp1*, *Arg1*, *Olr1*, *Ell2*, and *Trem1* (Figure 3D). Analysis of MPC-specific TGF-β–stimulated genes revealed 15 genes highly increased at day 7. These genes were important in the production of, attachment to, or reorganization of the extracellular matrix, including *Fn1*, *Col1a1*, *Col1a2*, *Col3a1*, *Col5a2*, *Timp1*, *Mmp14*, *Mmp2*, *Lox*, *Angptl4*, *Tpm1*, *Marcksl1*, *Acta2*, *Ltbp2*, and *Kif26b* (Figure 3E). Because increases in TGF-β isoforms and their receptors might suggest increased TGF-β–specific signaling, we also analyzed the gene expression of these elements in our scRNA-Seq data (Figure 4A). In the MPCs, none of the genes for TGF-β or their receptors were appreciably changed in expression levels across time (Figure 4A). Conversely, in the Macs there was increased expression of *Tgfb1*, *Tgfbr1*, and *Tgfbr2* at day 7, with no change in other TGF-β isoforms (2 or 3) or *Tgfbr3* (Figure 4A). In fact, the Macs demonstrated equal or greater TGF-β and receptor expression levels than the MPCs.

To get a better understanding of the genomic regulation of these TGF-β–stimulated genes in the Mac and MPC clusters, in a separate data set we performed single-nucleus ATAC sequencing (snATAC-Seq) on cells from the HO site of day 0 (uninjured) and 7 after injury. We evaluated the accessibility of chromatin around the known DNA binding sequence for p-SMAD2/3 in the genes for the TGF-β ligands and receptors. In the Macs, there was openness in promoter regions near SMAD2/3 binding sites for *Tgfb1*, *Tgfbr1*, *Tgfbr*2, corresponding to our scRNA-Seq findings (Figure 4B). The MPCs showed some increased openness in these genes at SMAD2/3 binding sites; however, the change was more prominent in the Macs (Figure 4B). Next, we analyzed chromatin accessibility at SMAD2/3 binding sites in the 15 Mac and 15 MPC TGF-β1–stimulated genes at day 7, identified above. In MPCs 12/15 genes and in the Macs 8/15 genes had open chromatin in SMAD2/3 binding sites in TGF-β1–stimulated genes identified (Supplemental Figure 4). Together these data support the histological data that canonical TGF-β signaling is occurring in both Macs and MPCs at the HO anlagen after injury with greater change in TGF-β ligand and receptor levels in Macs.

### Early HO site Macs display TGF-β ligand-receptor pairs.

**Figure 5:**
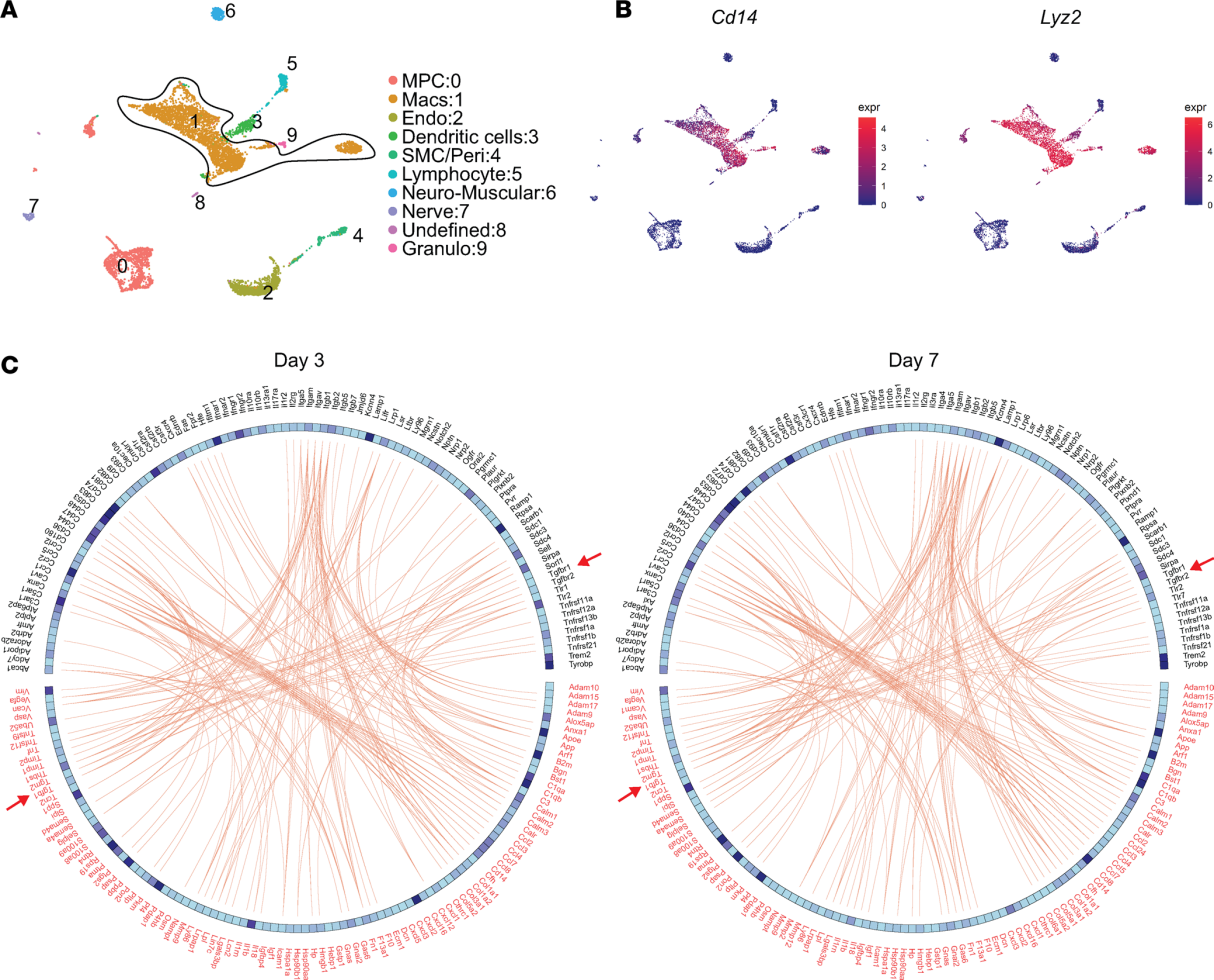
Ligand-receptor signaling in Macs. (**A**) UMAP plot of cells from day 0 and day 3 clustered with legend to the right of the plot. The composite Mac clusters are circled. (**B**) UMAP plots of genes marking Macs. (**C**) A list of ligand-receptor pairs was obtained from literature. The panel shows the top 100 expressed ligand-receptor pairs (red-black respectively), extracted from the Mac clusters at day 3 and day 7. Red arrows point to TGF-β1 signaling.

Based on our previous data, Mac infiltration into the HO site is at its peak 3 days after injury (10); therefore, we assessed cells at day 0 and day 3 postinjury from the HO anlagen and performed scRNA-Seq (GEO GSE126060). After sequencing, we identified 16 distinct clusters corresponding to known cell types, and we isolated the 3 clusters with Mac cell type into cluster 1, based on same expression markers mentioned above (Figure 5, A and B). These composite Mac clusters were used for subsequent analysis in addition to the canonical analysis with day 7.

To get a better understanding of the putative autocrine TGF-β signaling that might be occurring in these early infiltrating Macs, we performed receptor-ligand pairing analysis. To do this, a list of all potential ligands and receptors was adapted from humans to mice (41) and was compared against cellular features present in our scRNA-Seq data sets, of which 1,044 are applicable. The top 100 ligand-receptor pairs expressed in the Macs at our HO site on day 3 or 7 was subsequently used. This list was cross-referenced to confirm the expression of the ligands’ cognate receptors within the same Macs. This resulted in a list of 100 receptor-ligand pairs. Of these pairs, pathways associated with growth factor signaling, like TGF-β1, were identified (Figure 5C). The data suggest early Mac autocrine TGF-β signaling after traumatic injury plays a role in HO formation.

### TGF-β receptor type 1/ALK5 perturbation in MPCs and Macs.

**Figure 6:**
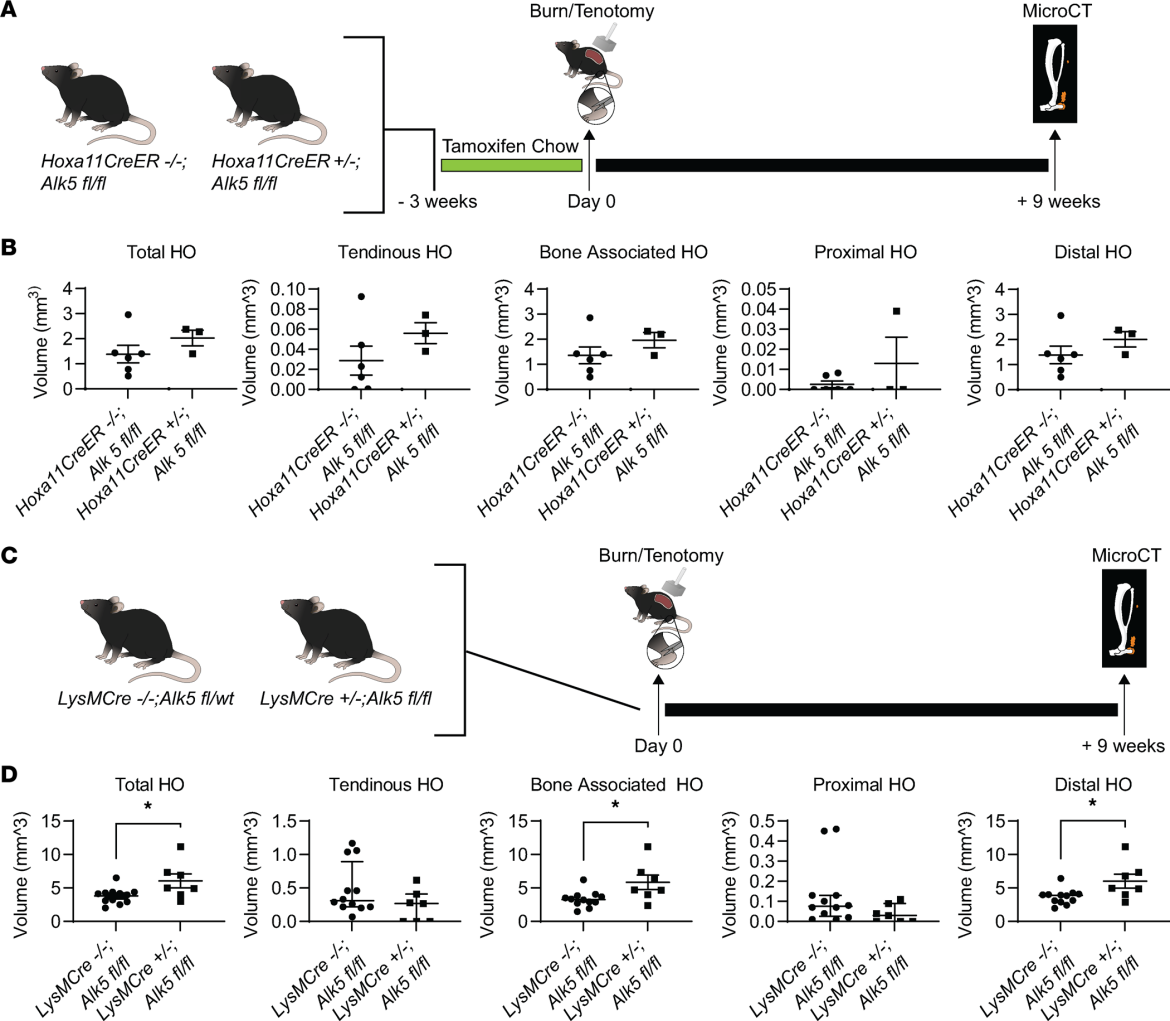
Loss of Alk5 signaling in Macs but not in MPCs has a greater impact on trauma-induced HO. (**A**) Graphic depicting experimental timeline for *Hoxa11CreER^T2^* mice. (**B**) MicroCT analysis of left injured hind limb 9 weeks postinjury for *Hoxa11CreER^T2–/–^ Alk5^fl/fl^* compared with *Hoxa11CreER^T2^ Alk5^fl/fl^* (*n* = 11 and 7, respectively/group). Error bars represent mean ± SEM. (**C**) Graphic depicting experimental timeline for *LysMCre* mice. (**D**) MicroCT analysis of left injured hind limb 9 weeks postinjury for *LysMCre^–/–^ Alk5^fl/fl^* and *LysMCre^+/–^ Alk5^fl/fl^* (*n* = 12, 7, respectively/group). Error bars represent mean ± SEM or median ± interquartile range (tendinous and proximal HO only). *P < 0.05 (the data were parametric, as explained in the Methods section).

We next set out to determine whether ALK5 signaling in Macs, MPCs, or both is important to HO formation after traumatic injury. To do this, we used a mouse with either myeloid or MPC-specific deletion of *Alk5*. To do this, we targeted the TGF-β1 receptor type 1, TGF-βRI, encoded by *Alk5*, using *Alk5^fl/fl^* mice. To delete *Alk5* in MPCs, we chose to cross our *Alk5^fl/fl^* mice with the *Hoxa11CreER^T2^* mouse line, creating *Hoxa11CreER^T2^ Alk5^fl/fl^* mice (42). Hoxa11 is a homeobox transcription factor expressed specifically in the zeugopod (radius/ulna, tibia/fibula). Although *Hox* genes are important in patterning during embryonic development (43), *Hoxa11* has been shown to mark MPCs throughout life (42). Work in our laboratory has demonstrated that these Hoxa11-expressing cells are those that form HO in our model (44). These mice allowed conditional deletion of *Alk5* only in the zeugopod region by administration of tamoxifen prior to inducing injury, thus avoiding adverse effects of *Alk5* deletion during development. MicroCT analysis 9 weeks after injury in the *Hoxa11CreER^T2^* *Alk5^fl/fl^* mice revealed no statistical difference in the amount of HO formed (Figure 6, A and B).

Next, we evaluated the role of ALK5 signaling in Macs, by crossing with the *LysMCre* mouse line (*LysMCre Alk5^fl/fl^*). We found that *LysMCre^+/–^ Alk5^fl/fl^* mice developed increased volume of HO compared with *LysMCre^–/–^ Alk5^fl/fl^* littermate controls (6.07 ± 1.03 mm^3^ 3.81 ± 0.34 mm^3^ (*P* = 0.02, *n* = 7 and 2, respectively). In our tenotomy model of HO, ectopic bone forms at 2 distinct anatomic sites: i) growing off the calcaneus (bone-associated HO) and ii) growing off the proximal cut end of the tendon (tendinous HO; Supplemental Figure 5A) Specifically, we found that *LysMCre^+/–^ Alk5^fl/fl^* mice developed increased bone-associated/distal HO compared with littermate controls (Figure 6, C and D). In contrast, we found that there was not an increase in tendinous HO and there was actually a decrease in this region (though not statistically significant; Figure 6D). We therefore, wondered whether this difference was due to the different Mac populations in tendon and bone. Bone-associated resident Macs, those marked by CD169 (45), might be important drivers of HO, whereas tendinous HO might be driven by circulating Macs. We confirmed by histology that CD169^+^ Macs, not thought to be marked by the *LysMCre* allele, were ALK5^+^ across multiple time points (days 0, 3, 7, and 21) in both the endosteum and periosteum (Supplemental Figure 5B). Therefore, we set out to block this TGFB/TGFBR signaling cascade through upstream blockade of the TGF-β1 and β3 ligands, which should affect both Mac populations.

### TGF-βRII-Fc treatment decreases early cavernous bone and mature bone in vivo.

We used a ligand trap (TGF-βRII-Fc) to determine if the bone-associated effect observed could be circumvented. Before use in vivo, we first tested the equilibrium binding constant of the TGF-βRII-Fc using surface plasmon resonance. Our results showed TGF-β1 and -β3 had a *K_D_* of 14.8 pM and 11.2 pM, respectively, compared with *K_D_* of 11,600 pM for TGF-β2 (Table 1). Subsequently, the IC_50_ was determined using an A549 luciferase reporter cell line for TGF-β signaling, including TGF-β1, TGF-β2, and TGF-β3. After the addition of the TGF-βRII-Fc, the IC_50_ was calculated to be 22.9 pM and 4.46 pM for TGF-β1 and -β3, respectively, but greater than 88,000 for TGF-β2 (Table 1). Overall, these data indicate the ligand trap has much greater affinity for binding TGF-β1 and -β3 with little effect on TGF-β2.

**Figure 7:**
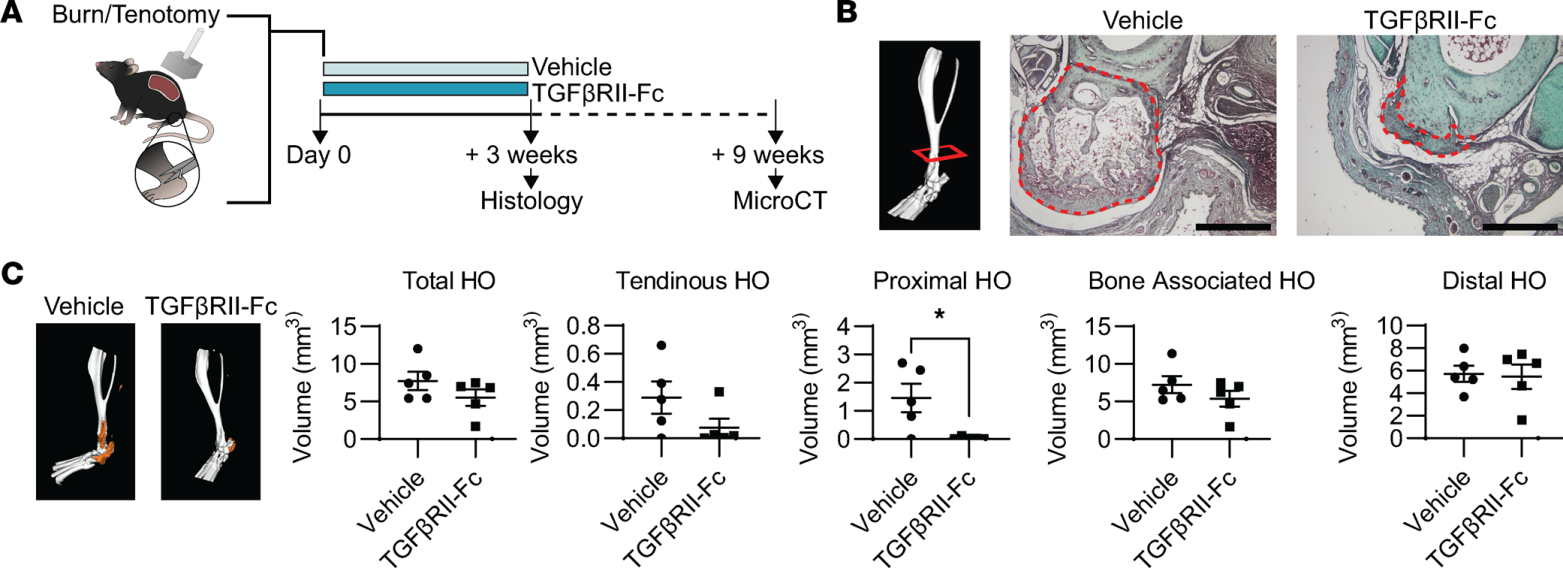
Effects of ligand trap (TGF-βRII-Fc) treatment on HO formation. (**A**) Graphic to depict model and experiment. To the right is graphic depicting HO formation by MicroCT and the regions assessed and what those include. (**B**) Example MicroCT image with red box indicating the approximate level histologic sections were taken from. Safranin O stains of vehicle- and ligand trap–treated hind limbs (*n* = 2/group). Region of HO is outlined in red. Scale bars represent 500 μm. (**C**) MicroCT reconstructions of representative samples where the HO is indicated in orange. Graphs of HO volume quantification with proximal HO showing significance by Student’s *t* test (*n* = 5/group). Error bars represent mean ± SEM for parametric data.

Next, to evaluate TGF-βRII-Fc treatment of trauma-induced HO formation, we used our mouse model for trauma-induced HO. After injury, mice were administered either vehicle or TGF-βRII-Fc (10 mg/kg; twice weekly) by subcutaneous injection for 3 weeks. We chose to treat these mice for the 3 weeks based on our previous paper using inhibitors of BMP signaling receptors, similar *Alk* gene family members, in our model of HO (46). Further, we chose subcutaneous injection as previous studies of antibody injection using this route of administration show that the antibody is taken up into the systemic circulation (47). HO sites were harvested at both 3 weeks for histology and 9 weeks for microCT analysis (Figure 7A). Safranin O staining, for glycosaminoglycans and cartilage formation, demonstrated decreased early cavernous bone formation in mice treated with TGF-βRII-Fc compared with vehicle (Figure 7B). Next, we analyzed mature bone at 9 weeks after injury by MicroCT and found there was decreased bone formation by 3D reconstruction in the TGF-βRII-Fc treatment animals, specifically proximal bone volume (0.03 ± 0.02 mm^3^ compared with vehicle 1.45 ± 0.51 mm^3^, *P* = 0.026; Figure 7C). HO trabecular volume and porosity were significantly decreased in the TGF-βRII-Fc–treated group (4.68 ± 2.33 mm^3^ to 1.28 ± 0.63 mm^3^, *P* = 0.016 and 0.45 ± 0.05 mm^3^ to 0.024 ± 0.04 mm^3^, *P* = 0.013, respectively; Supplemental Figure 6, A and B). Importantly, we found that there was no difference in tibial cortical thickness in TGF-βRII-Fc–treated mice (Supplemental Figure 6C). Treatment with TGF-βRII-Fc decreased bone formed away from the tendon injury site and to a lesser degree in the region of injury.

### TGF-βRII-Fc treatment does not overtly affect MPC canonical TGF-β1 signaling or proliferation.

**Figure 8:**
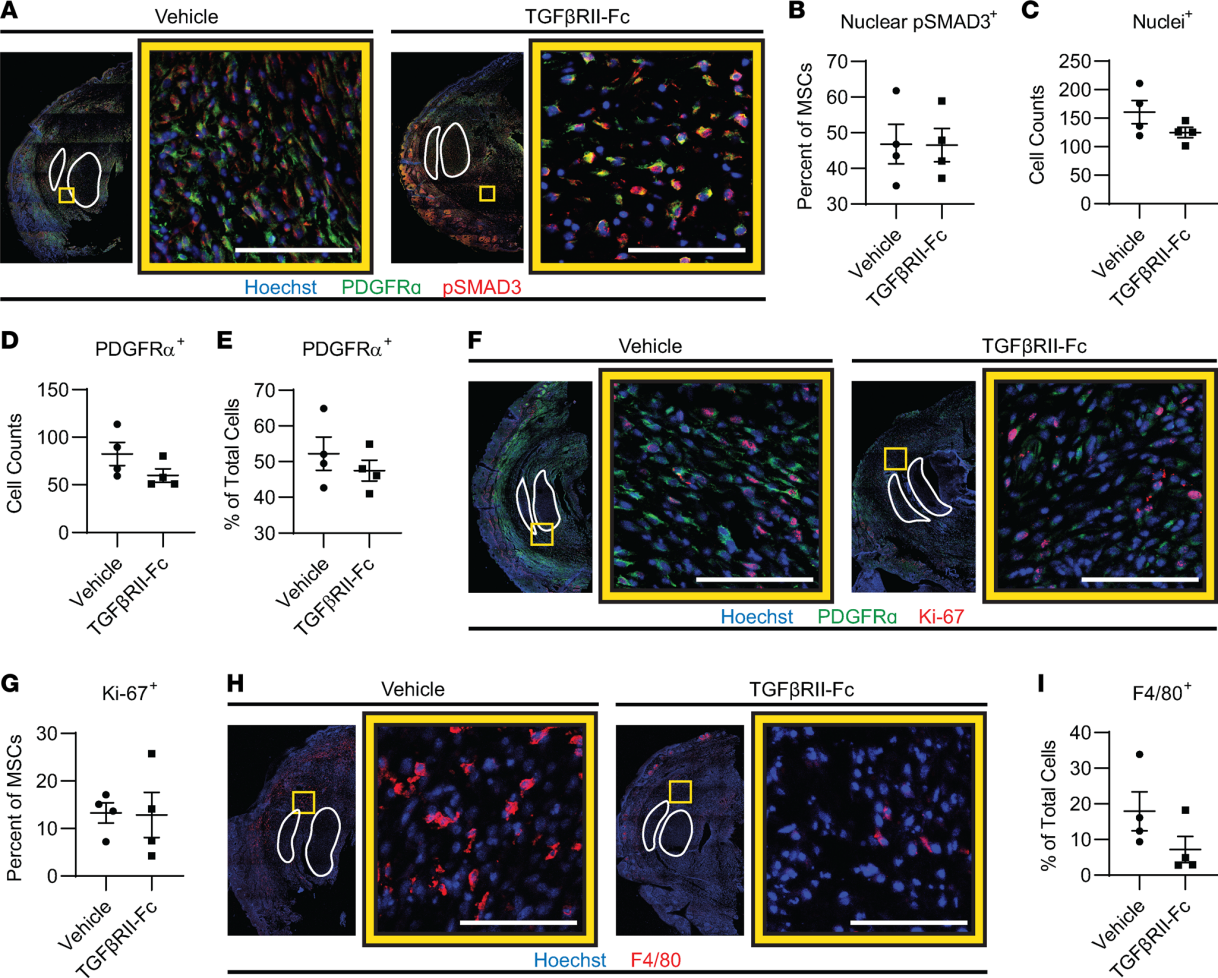
Ligand trap does not change in MSC canonical signaling or proliferation but trends toward decreased Macs. (**A**) IF tile scans of the distal hind limb where the tendons are outlined in white. The yellow box indicates the zoomed-in image location to the right of the tile scan where the 2 groups are identified above the images and the color legend is below. (**B**) Canonical TGF-β signaling by p-SMAD3 nuclear percentage in PDGFRα^+^ (MPCs) cells (*n* = 4/group, 3 images/*n*). (**C**–**E**) Further quantification from **A**, of the nuclei at injury site, total MPCs, and percentage of MPCs from total. (**F**) IF images and (**G**) quantification of proliferation by anti–Ki-67 in MPCs (*n* = 4/group, 3 images/*n*). (**H**) IF images and (**I**) quantification of percentage of Macs by anti-F4/80 (*n* = 4/group, 2–3 images/*n*). Error bars presented in graphs represent mean ± SEM for parametric data and represent median ± interquartile range for nonparametric data. Scale bars represent 100 μm.

Studies have shown that Macs lacking TGF-β receptors on their surface have both inhibited migration and M2 polarization (18). Therefore, we sought to determine by histology if the effects of TGF-βRII-Fc treatment were due to TGF-β canonical signaling on the Macs and not due to TGF-β signaling of the MPCs at the HO site. In support of this, we found no appreciable difference by IF in MPC TGF-β signaling (signified by percentage of PDGFRα^+^ cells that are nuclear p-SMAD3^+^) in the TGF-βRII-Fc–treated group compared to vehicle-treated controls (46.5% ± 4.7% vs. 46.8% ± 5.6%, *P* = 0.9671) (Figure 8, A and B). Further, quantification of the cell count by quantification of number of nuclei in the injury site demonstrated that there was a trend toward overall decreased cells in the TGF-βRII-Fc–treated group (160.7 ± 20.5 vs. 125.0 ± 9.1 cells, *P* = 0.1628) as well as a decrease in cell count of PDGFRα^+^ cells (82.3 ± 12.3 vs. 59.8 ± 6.9 cells, *P* = 0.1607) and a decrease in the percentage of total cells that were PDGFRα^+^ (52.2% ± 4.7% vs. 47.5% ± 2.9%, *P* = 0.4169; Figure 8, C–E). Analysis of proliferation by Ki-67 staining revealed the percentage of MPCs (PDGFRα^+^ cells) that were Ki-67^+^ was not different with TGF-βRII-Fc treatment compared with control (12.9% ± 4.8% vs. 13.3% ± 2.1%, *P* = 0.9385; Figure 8, F and G). Together, there are no appreciable changes in MPC TGF-β signaling or proliferation when treated with the ligand trap.

### TGF-βRII-Fc modulates injury site inflammation.

**Figure 9:**
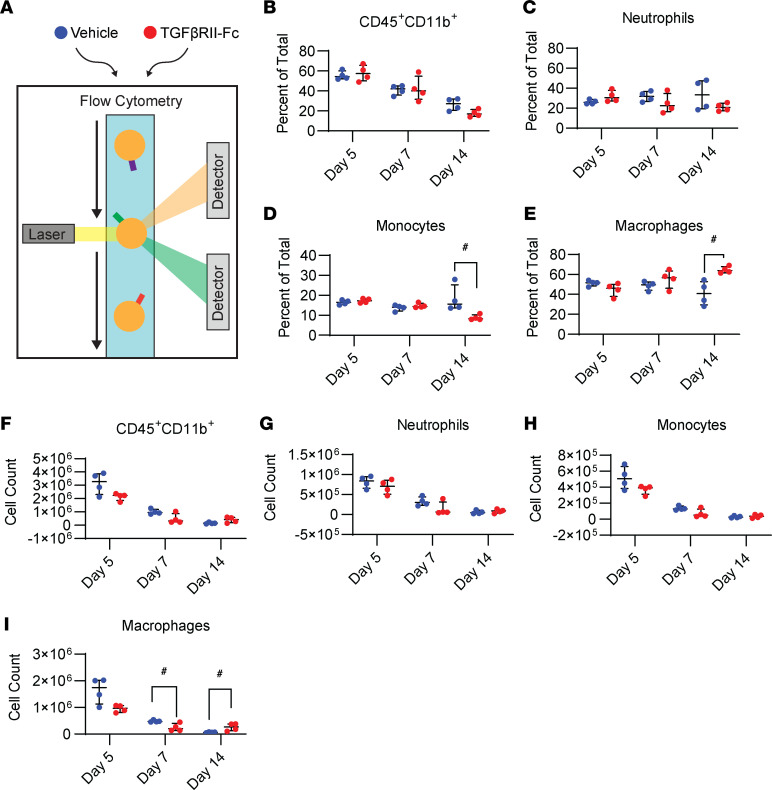
Flow cytometry shows treatment alters monocyte/Macs at damaged left hind limb tissue. (**A**) Graphical depiction of cell groups undergoing flow cytometry (3 time points, *n* = 4/group). (**B**–**E**) The percentage of total cells for CD45^+^CD11b^+^, neutrophils, monocytes, and Macs. (**F**–**I**) The total cell counts. Error bars presented in graphs represent median ± interquartile range for nonparametric data. ^#^*P* < 0.05 (the data were nonparametric, as explained in the Methods section).

TGF-β ligands are also known to be important drivers of immune cell recruitment during inflammation. TGF-β has been shown to stimulate chemotaxis of neutrophils and Macs (15, 48–52); therefore, we sought to determine if TGF-βRII-Fc treatment affected immune cell infiltration, particularly Macs, into the HO site. IF imaging of the HO site for the Mac marker F4/80 in TGF-βRII-Fc–treated or vehicle control mice 1 week after injury demonstrated that mice treated with TGF-βRII-Fc had a decrease in the percentage of F4/80^+^ cells (17.9% ± 5.5% vs. 7.2% ± 3.7%, *P* = 0.2000; Figure 8, H and I) at the HO anlagen. This suggests that TGF-βRII-Fc treatment acts by altering Mac migration to the site of injury. Further, flow cytometry of cells from the extremity injury in the treatment group and vehicle control for days 5, 7, and 14 (Figure 9A) showed decreased myeloid (CD45^+^CD11b^+^) cell counts and overall percentage of cells across time points, independent of treatment group (Figure 9, B and F). The change in myeloid cells after injury echoes previous reports (10). There was no significant change between the treatment groups in the percentage of neutrophils at each time point (Figure 9, C and G). However, there was a decrease in neutrophil total cell count numbers, a trend also seen with monocytes and Macs across time points (Figure 9, D, E, H, and I). The percentage of monocytes in the tissue at day 14 showed a significant decrease after treatment with the ligand trap (15.7% vs. 8.3%, *P* = 0.03), with a trend toward decreased monocyte numbers earlier with treatment (Figure 9, E and H). In the treatment condition, total Mac count was significantly decreased at day 7 (4,734,448 vs. 205,884 cell count, *P* = 0.03; Figure 9I). In summary, these data demonstrate that TGF-βRII-Fc modulates HO formation and Mac migration.

## Discussion

Traumatic HO is a debilitating and complex pathological process where endochondral ossification occurs secondary to injury and inflammation. MPCs are known to undergo aberrant differentiation in the development and progression of HO. Inflammatory cells, specifically myeloid cells, have also recently been shown to play a central role in this form of aberrant wound healing (9, 10). Prior studies have demonstrated that targeting Mac TGF-β expression can hamper the formation of HO (10). However, it is unknown whether TGF-β signaling via ALK5 exerts its effects in the Macs through an autocrine loop or on MPC differentiation via a paracrine role.

There is extensive literature on TGF-β ligands participating in wound healing, particularly in aberrant wound healing such as fibrosis and ectopic bone formation (10, 53–57). TGF-β1 is a master regulator after acute injury (53), interacting with almost every cell type involved. TGF-β1 has been shown to lead to fibroblast migration (3) and activation (58) into an injury site. Elevated TGF-β1 at a wound site results in recruitment of circulating inflammatory cells, such as neutrophils and Macs (53). Macs, in turn, migrate to the wound site and secrete cytokines, including more TGF-β1. Macs with an antiinflammatory, immune-suppressive, proangiogenic, and proregenerative phenotype, classified as M2, are known to produce TGF-β1; TGF-β1 signaling in the Mac itself has been suggested to play an important role in polarization to this “alternate” phenotype (18). Here we show increased TGF-β downstream signaling in MPCs and Macs at the HO site, changes in expression of TGF-β ligands and receptors, and changes in chromatin accessibility. With the addition of ligand-receptor pair analysis in early infiltrating Macs, we suggest that TGF-β is playing an autocrine role.

Selective genetic deletion of ALK5 in *LysMCre^+^* myeloid cells increased bone-associated HO. The increased HO pattern in this genetic model is inconsistent with the results of systemic TGF-βRII-Fc administration where there is a decrease in proximal HO, located at the retracted proximal tendon stub status after tenotomy. The same proximal pattern is seen in our prior study in which TGF-β1 was altered in Macs genetically or with CD47 activating peptide (10). Unlike the proximal HO pattern, the distal pattern did not present in our previous study. It is possible the HO increase in *LysMCre Alk5^fl/fl^* mice could be a result of CD169^+^ bone Macs, distinct from osteoclasts. Bone Macs are known to affect osteoblast differentiation and bone mineralization (59). The Macs might not express *LysMCre* and thus retain the receptor, and ultimately have unmitigated TGF-β signaling (45). Alternatively, the TGF-β receptor complex is heterodimeric receptor consisting of 2 types (TGF-βR1 and TGF-βR2), and there is evidence that loss of TGF-βR1 (ALK5) could have ongoing signaling through TGF-βR2, such that loss of both receptor types is necessary to completely stop TGF-β signaling (60, 61). Another possibility is that the loss of *Alk5* in Macs also alters their migration and polarization so profoundly that they are unable to become a more regenerative Mac, which has some salubrious effects to limit the bone-associated HO. Future studies using more flow and newer modalities, such as spatial transcriptomics, might be able to provide clarity on if there are population differences spatially in the regions of HO anlagen. We decided to focus less on the pathophysiology of this unintended effect and more on finding a way to altogether bypass the receptor signaling by altering signaling at the ligand level, which is why we utilized a ligand trap (TGF-βRII-Fc) with the added benefit of being a more feasible treatment option.

With the ligand trap (TGF-βRII-Fc) treatment, we noted decreased proximal/tendinous HO. Our IF and flow cytometry data looking at the HO anlagen site 1 week after injury demonstrated there were decreased monocyte/Macs at the site with ligand treatment, suggesting a delay in monocyte/Mac migration to the injury site. Though not statistically significant, a similar decreasing trend of HO was seen in the *LysMCre Alk5^fl/fl^* mice. Previous research has shown inhibition of ALK5 alone can impair monocyte migration toward TGF-β1 (52). Our findings support that the trend toward decreased tendinous HO noted in the *LysMCre Alk5^fl/fl^* mice is also due to impaired monocyte/Mac migration.

It is well documented in the literature that TGF-β1 is an important factor in chondrogenesis (62), cartilage, and joint formation (63). In fact, the effects of TGF-β on MPCs has been shown to be complex and context dependent. Latent and soluble forms of TGF-β through alternative pathways can drive human mesenchymal stem cells to chondrogenesis (64). Studies have shown that the latent form signals through an integrin-mediated pathway (65), though others have shown there are mechanotransducive effects, such as the ROCK pathway, that augment the signaling of TGF-β (66). Additionally, hypoxia plays a role in signaling (67).

Given how the literature is so demonstrative toward TGF-β’s prochondrogenic effects, it raises the question of why in our study the loss of TGF-βRI signaling specifically in MPCs resulted in no significant change in HO formation after traumatic injury. Data presented by Wang et al. where TGF-βRI functions in cartilage to block BMP signaling in resting growth plate chondrocytes do support our findings (60). Therefore, in our MPC-specific deletion mice, chondrocytes formed from MPCs would not have BMP signaling inhibited by TGF-βRI signaling, and this drives the formation of HO. Additionally, it has been shown that deletion of both TGF-βRI and TGF-βRII is necessary for complete signaling inhibition in TGF-β1 signaling (61, 68).

Currently, effective management of HO is limited, with prophylactic NSAIDs or radiation therapy offering only modest benefit (69). Surgical excision can be done but with substantial chance of recurrence. For example, in elbow HO there is around 20% recurrence following surgical excision (70). To expand and improve treatment options, we need to understand the underlying signaling pathways to find potential targets for therapy. Mouse models have shown a nonselective TGF-β neutralizing antibody attenuates HO formation in a tendon puncture model (9). A primate HO model suggests aberrant bone is mediated by TGF-β1, and in human HO tissue there are elevated levels of TGF-β, suggesting the role of TGF-β ligands on HO formation is conserved across species (9, 71). Therefore, therapeutic targeting of TGF-β ligands has the potential to mitigate the burden of HO.

We demonstrate that Macs are the critical target of TGF-βRI signaling for aberrant wound healing after traumatic injury. We also show that systemic treatment with TGF-βRII-Fc modulates TGF-β signaling upstream of the TGF-βRI, impairing monocyte/Mac migration, such that HO formation is attenuated, particularly in the proximal region. These data signify that targeting TGF-β ligands and Mac autocrine signaling after traumatic injury is an effective future therapeutic target to improve wound healing and prevent aberrancies such as muscle fibrosis (54) or HO.

## Methods

### Mouse use and treatments.

Mice were housed in standard conditions. All animals used were C57BL/6 background mice. C57BL/6 mice were purchased from The Jackson Laboratory (000664). All mice received preoperative and 48 hours postoperative subcutaneous buprenorphine (0.06 mg/kg) for analgesia. Animals were anesthetized with inhaled isoflurane. Mice received 30% total body surface area partial-thickness dorsal burn. The dorsal burn was induced using a metal block heated to 60°C in a water bath and applied to the dorsum for 18 seconds continuously. Tenotomy was performed by transection of the left Achilles tendon. Animals were assigned to the vehicle control or ligand trap group. Vehicle control was 1× PBS and ligand trap was muTGFbRII-mFc, shortened to TGF-βRII-Fc, supplied by Acceleron Pharma, Inc. Treatment began the day of surgery (day 0). Mice were administered vehicle or ligand trap (10 mg/kg) subcutaneously twice weekly for 3 weeks. Mice were euthanized for experiments at 1, 3, or 9 weeks after injury for experimental analysis.

*Hoxa11CreER^T2^* and *LysMCre* were bred in-house with *Alk5^fl/fl^*. The *Hoxa11CreER^T2^* line was obtained from Deneen Wellik at the University of Wisconsin, Madison, Madison, Wisconsin, USA. These were then crossed with the *Alk5^fl/fl^* line that was obtained from Katherine Gallagher at the University of Michigan to produce *Hoxa11CreER^T2^ Alk5^fl/fl^* mice. To induce the deletion of *Alk5*, *Hoxa11CreER^T2^ Alk5^fl/fl^* mice were placed on tamoxifen chow for 3 weeks when they were 5 weeks of age. *LysMCre* mice were crossed with *Alk5^fl/fl^* mice, both from The Jackson Laboratory, to generate *LysMCre Alk5^fl/fl^*. Littermates of both crosses were used as controls. Mice underwent the burn/tenotomy injury described above. Legs were harvested at 9 weeks and MicroCT analysis was performed. *CD169Cre* mice were obtained from Riken Group (RBRC06239) (72, 73). *CD169Cre* mice were crossed with *Rosa26-tdTomato* (Deneen Wellik, supplemented with The Jackson Laboratory strain 007905) to obtain double-heterozygous mice. They underwent our injury model and tissue harvested for IF histology.

### scRNA-Seq analysis.

Single-cell data replicates from day 0 (uninjured), 3, 7, and 21 postinjury were taken from GEO GSE126060 (10). We considered cells and genes as per our previous analysis (44). Briefly, we selected replicates to have a consistent number of cells for each time point: day 0 (replicates 1–4, 3,815 cells), day 3 (replicates 1–2, 4,201 cells), day 7 (replicate 1, 3,405 cells), and day 21 (replicates 1–3, 3,505 cells) for multiple–time point analyses (43). Cells were retained based on genes expressed in more than 10 cells (17,131 genes), total expressed genes in the range of [500, 5,000], and the fraction of mitochondrial gene unique molecular identifiers lower than 0.2. Counts were normalized (default parameters) and scaled (regressing against number of genes expressed per cell and fraction of mitochondrial expression). Variable genes were extracted (default Seurat parameters) and defined as the intersection of the top 5,000 genes for each replicate (1,497 genes). Replicates were joined via canonical correlation analysis (20 components, using the overall variable genes). Sixteen cell populations (numbered 0 to 15) were obtained with Louvain algorithm, resolution 0.4, using the aligned canonical correlation components. FindMarkers (default Seurat parameters) was utilized to extract the markers from each population. Markers were ranked according to the difference in the fractions of cells expressing each marker within the population versus rest of the considered cells. Top markers were used to label the cell populations.

### Multiple–time point analyses.

Two analyses were set up in Seurat 3.1.4 (73), considering 3 time points (0, 7, and 21) and 2 time points (0 and 3). Each analysis consisted of subsetting genes, cell counts, and cell identities (clusters) from the joined set described above. For each analysis, the subset data were merged. After merging, data were processed by normalization, identification of variable genes, scaling, dimensional reduction (principal component analysis), and nonlinear dimensional reduction (UMAP). The first 15 dimensions were used for the UMAP, while default parameters were kept for all the other steps. The 16 cell populations from prior analysis (44) were then merged and labeled as 10 final clusters according to their identity. More specifically, we merged multiple Mac clusters (clusters 1, 2, 4), mesenchymal clusters (clusters 0, 6, 8), endothelial clusters (clusters 2, 5), and pericyte/smooth muscle clusters (clusters 9, 11). The MPC cluster as well as the Mac cluster were used for subsequent analysis. Individual genes were assessed in MPC or Mac clusters for expression levels across time points. Gene expression markers for MPC cluster or Mac cluster were generated in comparison to the rest of cells/clusters for each time point. Gene expression markers for MPC cluster or Mac cluster were generated in comparison with the rest of the cells/clusters for each time point based on prior analysis by our group (43). Individual genes were assessed in MPC or Mac clusters for expression levels across time points.

### Mac ligand-receptor pair analysis.

To perform receptor ligand pairing analysis on our Mac cluster, a list of all potential ligands was adapted from a human ligand-receptor database (44) for mice. There were a total of 1,372 unique mouse genes for ligands and receptors. Of these, 1,044 were expressed in our Mac cluster. We considered days 3 and 7 independently. Ligand and receptor genes were ranked according to the average counts per cell in the Mac cluster. The ligand-receptor pairs including the top 100 ranked ligands and receptors were used to create circle plots.

### snATAC-Seq analysis and genome tracks.

snATAC-Seq was performed using Signac 3.1.5 (https://github.com/timoast/signac; commit ID fa23843) as previously described by our group using GEO GSE150995 (44). Data from day 0 and day 7 were combined after filtering the data set to cells that have at least 100 features. The combined Seurat object was then normalized using the default set of parameters, and top variable peak accessibilities were calculated using a cutoff of at least 20 cells. Dimension reduction was done using t-distributed stochastic neighbor embedding with dimensions 2 through 30 used as input features. A shared nearest neighbor modularity optimization-based algorithm with a resolution of 0.2 was used to determine unique clusters. Clusters from scRNA-Seq data were used to guide the labeling of clusters using the FindTransferAnchors function. The MPC cluster of cells was isolated, and BigWig files were generated for each day using sinto (https://timoast.github.io/sinto/) and deeptools (74). BigWig files were uploaded into the open source software Integrated Genomics Viewer (75, 76). The data range for the open chromatin tracks (day 0 and 7, Mac cluster, and 2 MPC clusters) was set from 0 to 800 across all tracks. The SMAD binding element was input to evaluate motif (77). Genes were assessed for open chromatin in promoter regions.

### Surface plasmon resonance analysis.

All analyses were performed with a Biacore T100 instrument (GE Healthcare, now Cytiva). An anti-mouse Fc-specific antibody was immobilized on a Series S CM5 sensor chip through amine coupling by following the manufacturer’s instructions (GE Healthcare). HBS‑EP buffer (GE Healthcare) supplemented with 350 mM NaCl and 0.5 mg/mL bovine serum albumin (BSA) was used as running buffer. TGF-βRII-Fc was captured on the experimental flow cell at a density of approximately 50 response units. TGF-β1 and TGF-β3 (0.013–10 nM) and TGF-β2 (0.04–90 nM) were injected in 3-fold serial dilution over the captured protein for 300 seconds, followed by 600 seconds dissociation time at a flow rate of 70 μL/min with buffer blanks injected periodically for double referencing. The chip regeneration was performed with 10 mM glycine pH 1.7. All sensorgrams were processed by double referencing (subtraction of the responses from the reference surface and from an average of blank buffer injections). To obtain kinetic rate constants, TGF-β1 and TGF-β3 data were fit to 1:1 interaction model that includes a term for mass transport using BIAevaluation software (GE Healthcare). A concentration range of 0.013–1.1 nM for both TGF-β1 and TGF-β3 was used to fit TGF-βRII-Fc binding sensorgrams. The equilibrium binding constant *K*_D_ was determined by the ratio of binding rate constants *k_d_/k_a_*. Due to the transient nature of binding, the equilibrium binding constant *K*_D_ of TGF-β2 was determined by using the steady-state affinity model, where the maximal measured signal (in RU) before the end of the association phase (which is close to a plateau) is plotted against the ligand concentration, which ranges from 0.12 nM to 90 nM for TGF-β2 binding.

### Cell-based assays.

The cell-based assay utilizes A549 cells (human lung carcinoma cell line, CCL-185, ATCC). A549 cells were seeded in 48-well plates at 6.5 × 10^4^ cells per well in F-12K medium (ATCC) supplemented with 10% FBS and incubated overnight. All incubations were at 37°C, 5% CO_2_, unless otherwise noted. The cells were transiently cotransfected with both the luciferase vector pGL3 (CAGA)_12_ in which the firefly luciferase gene expression is under the control of the TGF‑β ligand-responsive (CAGA)_12_ element, and the constitutively active renilla luciferase plasmid pRL-CMV (Acceleron Pharma, Inc.) to normalize for transfection efficiency. This was done by combining 10 μg pGL3(CAGA)_12_, 0.1 μg pRL-CMV, and 30 μL XtremeGENE9 (Roche) with 970 μL Opti-MEM (Thermo Fisher Scientific) and incubating the mixture for 30 minutes at room temperature prior to adding 24 mL of assay buffer (F-12K (ATCC) supplemented with 0.1% BSA) and applying to the plated cells (500 μL/well) for an overnight incubation. The next day, 3-fold serial dilutions of TGF-βRII-Fc were made in assay buffer in a separate 48-well plate (8 data points starting at 100 ng/mL for TGF-β1 assay, 25,000 ng/mL for TGF-β2 assay, or 75 or 100 ng/mL for TGF-β3 assay). The final concentration of TGF-β1, TGF-β2, or TGF-β3 added to the corresponding wells in this plate was 640 pg/mL, 480 pg/mL, and 270 pg/mL, respectively. The receptor-ligand mixture was incubated for 30 minutes prior to applying 500 μL/well to the transfected A549 cells. The cells were harvested after an 18- to 20-hour incubation and assayed using the Dual Luciferase Reporter Assay system (Promega, with a Tecan Infinite M200 instrument) to determine normalized luciferase activity expressed as relative luciferase units.

### Histology.

Legs were decalcified, embedded, and sectioned as previously described by our lab (78). Safranin O staining (MilliporeSigma) was done on tissue cross sections from 3 weeks after burn/tenotomy. Bright-field images (Olympus BX-51) of cross sections were obtained (*n* = 2).

### IF.

IF was done as previously described (10). Briefly, sections were washed in 1× TBS with 0.05% Tween 20 (TBST), then incubated in donkey block at room temperature for 1–2 hours, and then primary antibodies in antibody diluent were applied and incubated at 4°C overnight. Primary antibodies were washed off with TBST. Slides were incubated with donkey secondaries for 1 hour at room temperature. Subsequently, slides were washed with TBST, counterstained with Hoechst, and mounted with ProLong Glass Antifade Mountant (Invitrogen, P36980). Details regarding antibodies and stains can be found in Table 2. IF images were obtained with Leica SP8 confocal inverted microscope or Leica STELLARIS 8.

### Microscopy image quantification.

Image area quantifications were performed in FIJI (79). The p-SMAD3 percentage area was obtained first by converting 20× original magnification images of the p-SMAD3 channel to 8-bit. Intermodule threshold was applied to encompass positive signal. Results were measured and exported to Microsoft Excel. All cell counts were performed by hand within the LASX software on either 20× or 63× zoomed-in images and exported to Excel for subsequent calculations.

### MicroCT.

Left legs were scanned using Bruker SkyScan 1176 MicroCT. Bone volumes were determined utilizing MicroCT imaging (35 μM resolution, 357 μA beam energy, 70 kV beam current, 520 ms exposure). Scans were analyzed using calibrated imaging protocol as previously described by MicroView micro CT viewer (GE Health Care and Parallax Innovations) (80). Bone reconstructions depicting representative means of treatment groups were calculated at 800 Hounsfield Units (HU). Ectopic bone was manually splined and measured at 0, 800, and 1,250 HU thresholds. Ectopic bone volumes were characterized as total volume, proximal, distal, bone associated, and tendinous.

### Flow cytometry.

Following a burn tenotomy injury, at time points of 5, 7, and 14 days, the soft tissue from the posterior compartment between the muscular origin and the calcaneal insertion of the Achilles tendon was dissected out and collected for processing. The tissue was digested for 20–30 minutes in 0.3% type 1 collagenase and 0.4% dispase II (Gibco) in Roswell Park Memorial Institute (RPMI) medium at 37 °C under constant agitation at 180 rpm. The digestions were quenched with 10% FBS in RPMI and then filtered through 40 μm sterile strainers. Specimens were blocked with anti-mouse CD16/32 and subsequently stained using the antibodies Ly6G, CD11b, Ly6C, and/or CD45 listed in Table 2. Samples were washed with FACS buffer (2% FBS in PBS), and then flow cytometry data were collected using a FACSCanto (BD). Analysis was performed using FlowJo software.

### Statistics.

For microscopy quantifications, statistical analyses were performed in GraphPad Prism 8, where associated graphs were also generated. Shapiro-Wilk was applied to assess for normality. Student’s 2-tailed *t* test was used for parametric data, and data are represented by mean ± SEM. Mann-Whitney *U* test was performed on nonparametric data and represented as median ± interquartile range. For MicroCT, statistical analyses were performed in IBM SPSS Statistics 24 and GraphPad Prism 8. Graphs were created in GraphPad Prism 7 or Microsoft Excel. Shapiro-Wilk test was used to determine the appropriate test. Two-tailed independent Student’s *t* tests were performed on parametric data at α = 0.05, with *P* value significance indicated by *. Mann-Whitney *U* test was performed on nonparametric data at α = 0.05, with *P* value significance indicated by ^#^. One-way ANOVA with Brown-Forsythe test for correction as well as Dunnett’s multiple comparison was performed on data at α = 0.05.

### Study approval.

All animal experiments described were approved by the Committee on the Use and Care of Animals at the University of Michigan, Ann Arbor (PRO0007930), and University of Texas Southwestern, Dallas (2020-102949). All animal procedures were carried out in accordance with the guidelines provided in the *Guide for the Use and Care of Laboratory Animals: Eighth Edition* (National Academies Press, 2011).

Prior publication: NKP, MS, CDH, SL, RK, and BL are authors on an abstract submitted to Plastic Surgery Research Council 63rd Annual Meeting, May 19, 2018; Birmingham, Alabama, USA; for which a small portion of the preliminary data was part of the abstract.

## Author contributions

NKP was responsible for conception and design, collection and/or assembly of data, data analysis and interpretation, and manuscript writing. JHN and MS were responsible for conception and design, collection and/or assembly of data, and data analysis and interpretation. SM was responsible for single-cell data analysis and interpretation and manuscript reading and editing. CAP and ALS were responsible for data analysis and interpretation and manuscript writing. CDH was responsible for data analysis and interpretation. SL was responsible for conception and design. KRP was responsible for snATAC-Seq analysis and pathway analysis. RK was responsible for conception and final approval of the manuscript. ACB, JAG, RN, HAR, and NL were responsible for data analysis and interpretation. KV was responsible for data analysis and study design. AKH was responsible for conception and design, data analysis and interpretation, collection and/or assembly of data, manuscript editing, and final approval of the manuscript. BL was responsible for conception and design, data analysis, and final approval of the manuscript.

## Supplementary Material

Supplemental data

## Figures and Tables

**Table 2:**
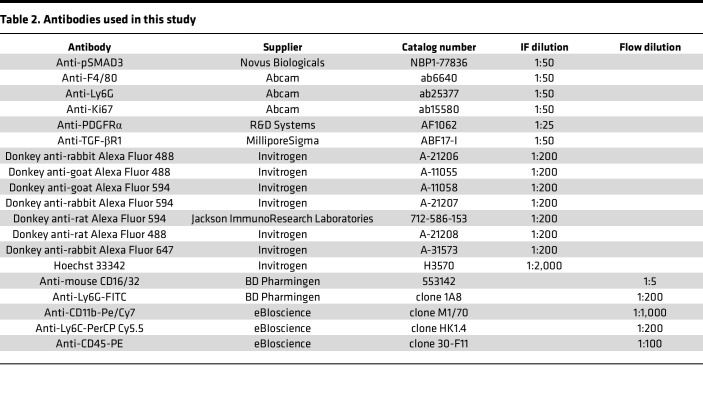
Antibodies used in this study

**Table 1:**
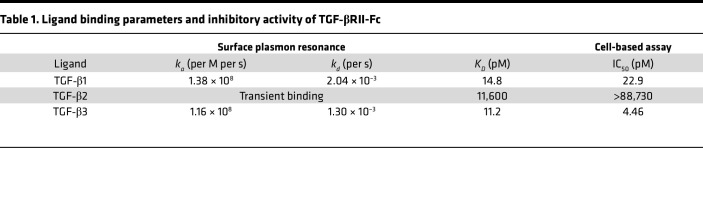
Ligand binding parameters and inhibitory activity of TGF-βRII-Fc

## Version 1 09/13/2022

In-Press Preview

## Version 2 10/24/2022

Electronic publication
