# Supplementary material for: Macrophage TGF-β signaling is critical for wound healing with heterotopic ossification after trauma
Source: JCI Insight. 2022 Oct 24;7(20):e144925. doi: 10.1172/jci.insight.144925 (PMC9714796; doi:10.1172/jci.insight.144925)
Supplement: Supplemental data [file jciinsight-7-144925-s185.pdf]

Supplemental Data:

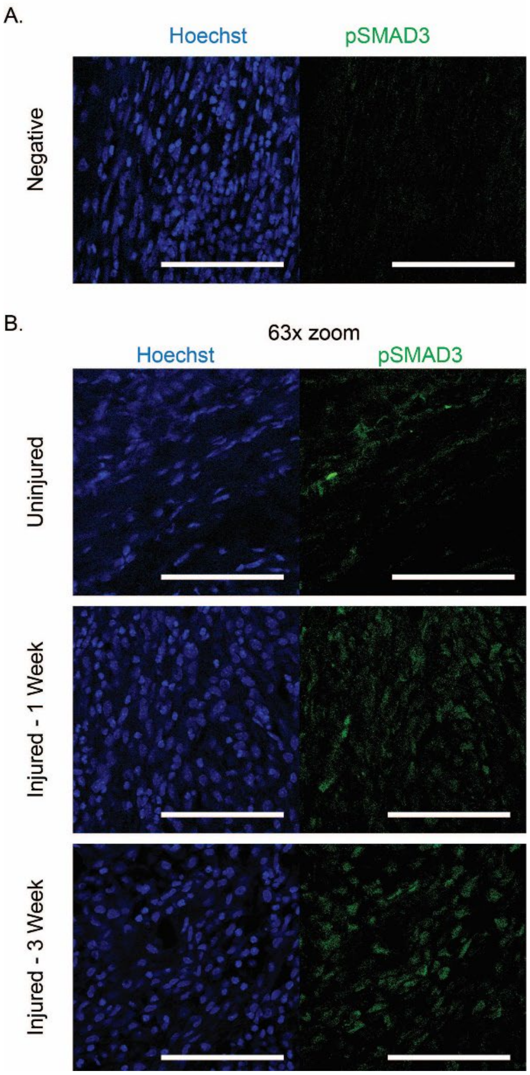

Supplementary Figure 1. Full uncropped IF images from Figure 1 with each channel separated. (A) Negative control with pseudocolored channels representing nuclei (blue) and pSMAD3 (green). **(B)** The images from uninjured, injured 1 week, and injured 3 week. Scale bars represent 100 µm.

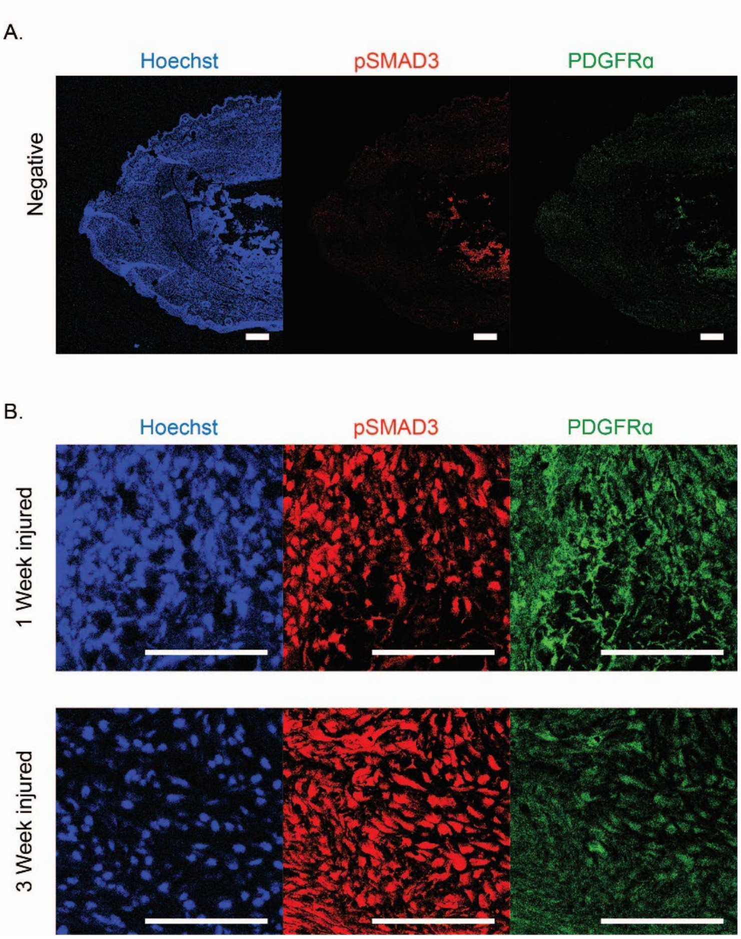

Supplementary Figure 2. Full uncropped IF images from Figure 2 A-B with each channel separated. **(A)** Negative control with pseudocolored channels representing nuclei (blue) and pSMAD3 (red) and PDGFRα (green). **(B)** The channel images from injured 1 week and injured 3 week are shown. Scale bars represent 100 µm.

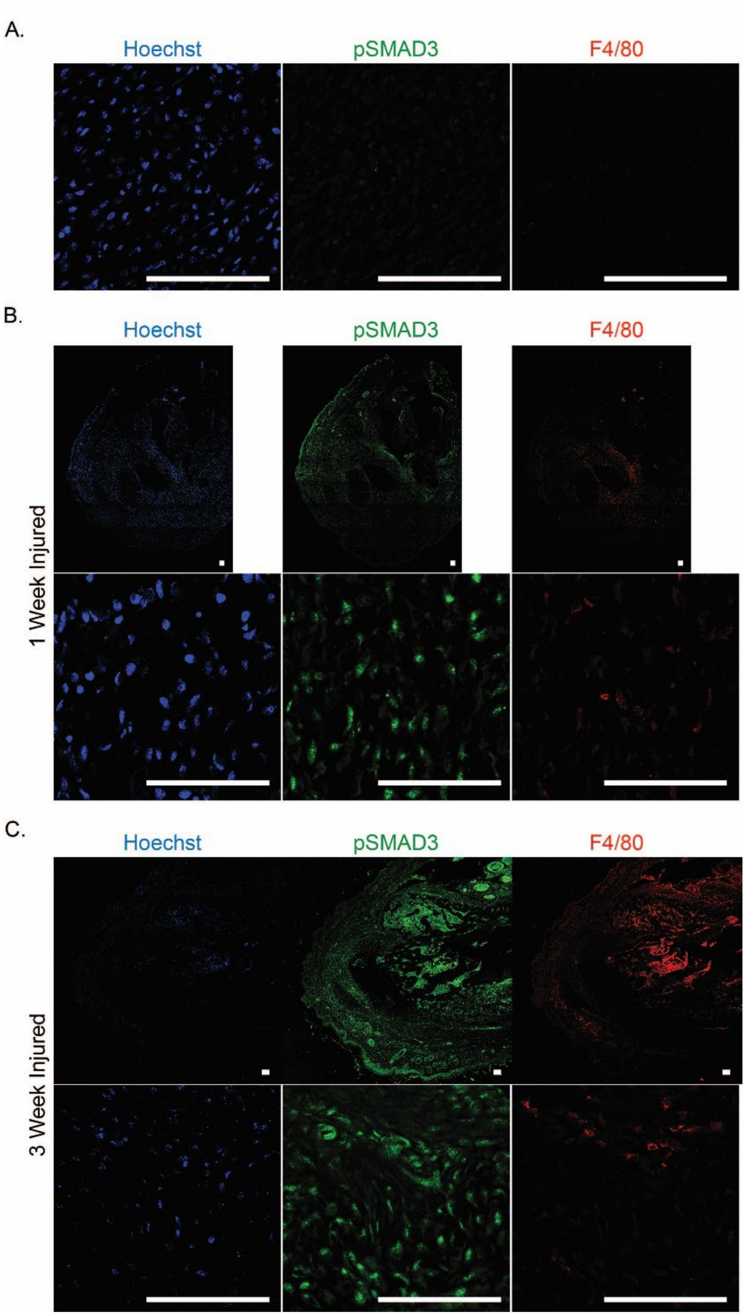

Supplementary Figure 3. Full uncropped IF images from Figure 2 D-E with each channel separated. **(A)** Negative control with pseudocolored channels representing nuclei (blue), pSMAD3 (green), F4/80 (red). **(B)** Channel images from 1 week post injury consisting of tile scan and 63x zoom. Also from **(C)** 3 week post injury. Scale bars represent 100 microns.

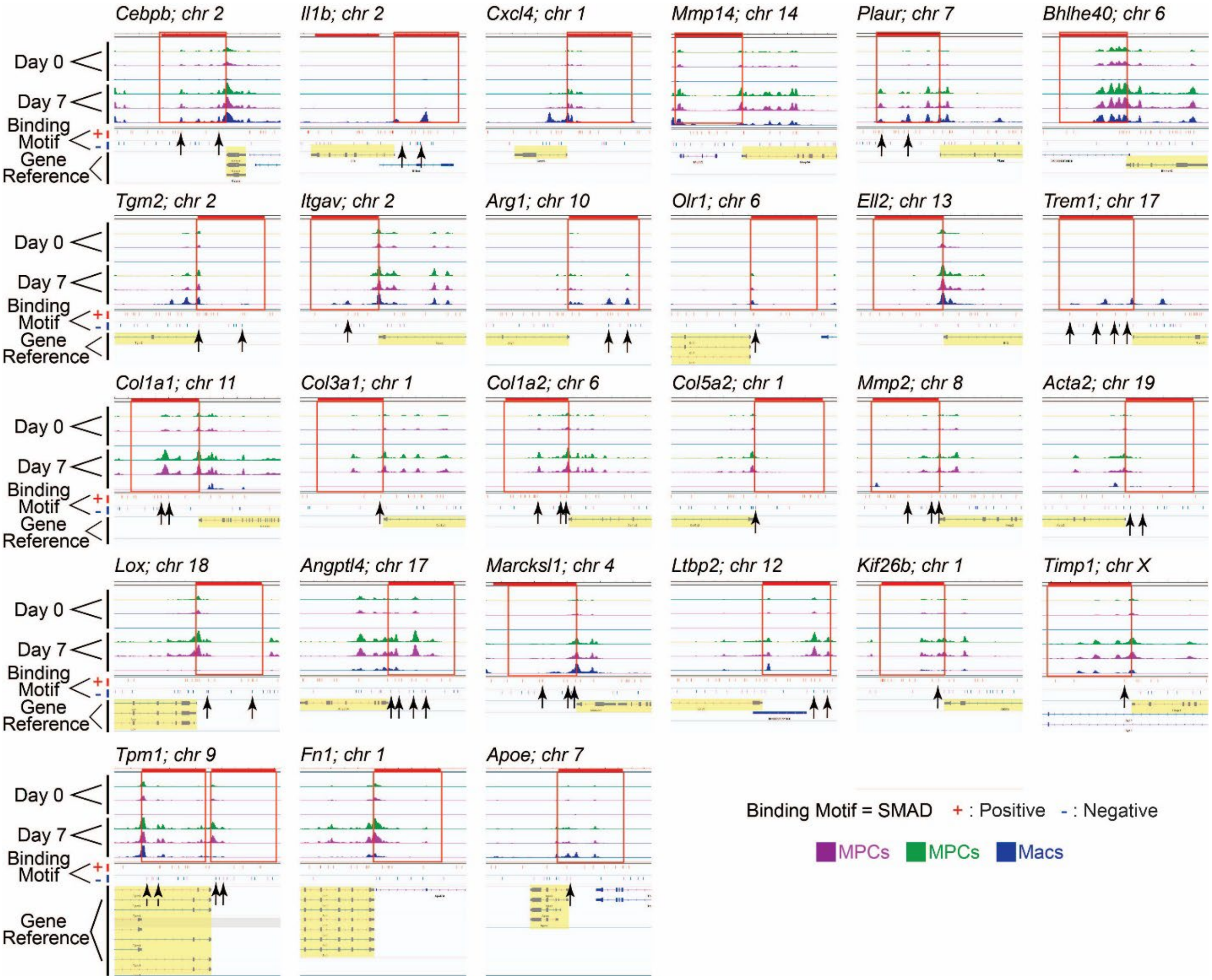

Supplementary Figure 4. Open chromatin in TGFβ stimulated genes and SMAD binding motif. Twenty-seven genes stimulated by TGFβ and found to be upregulated in Macs, MPCs, or both defined by the legend. Genes are highlighted yellow with a red box indicating ~5kb upstream promoter region. The tracks shown are color coded by their cluster identity such that green and magenta are MPCs and blue are Macs. All track data is presented in the range of 0 to 800. Black arrows are used to assist in indicating the regions of open chromatin at or near SMAD binding sites.

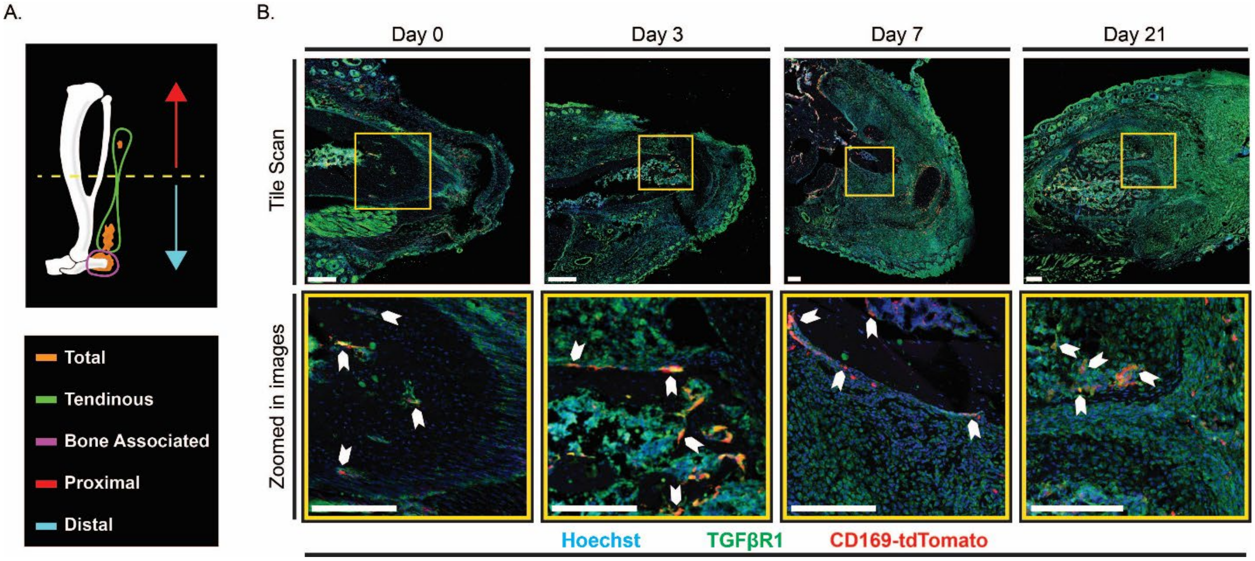

Supplementary Figure 5. Regions of MicroCT analysis and TGFβR1 expression in resident macrophages. (A) Graphic depicting the regions of HO. **(B)** There are macrophages present in the bone that express CD169 and TGFβR1 by IF. Top row showing merged tile-scans of day 0, 3, 7, and 21 (n=3, 1, 2, 1 per day at respective timepoints) with bottom row showing zoomed region of interest that corresponds to the yellow box. White chevrons are pointing to cells (nuclear Hoechst staining = blue) that are positive for both CD169 (red) and TGFβR1 (green). Scale bars represent 200 microns.

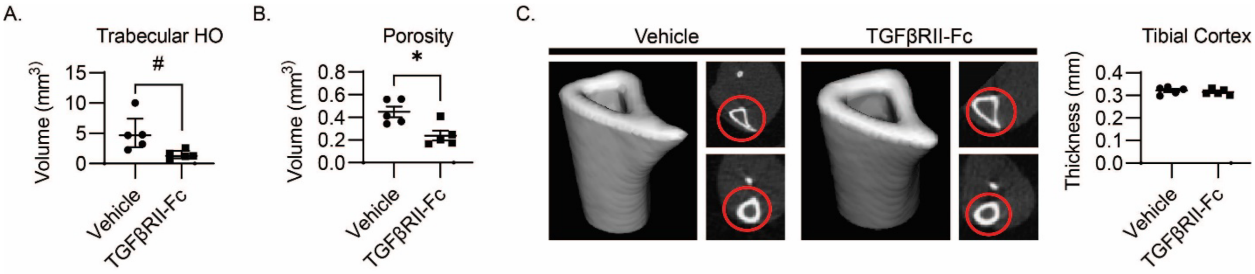

Supplementary Figure 6. Differences in HO bone features without change in native bone cortex. **(A)** Graph of trabecular HO volume and statistical significance set by Mann-Whitney U-Test. **(B)** Graph of HO porosity and statistical significance set by students t-test. **(C)** 3D reconstruction of tibia and associated cross-sections right of it, for vehicle and ligand trap treated samples. Graph to the right of images indicating cortical thickness in mm. **(A-C)** Error bars for all graphs are represented by mean ± SEM for parametric data or median ± quartile for non-parametric data (n=5/group).
